# Prevalence of workplace violent episodes experienced by nurses in acute psychiatric settings

**DOI:** 10.1371/journal.pone.0211183

**Published:** 2019-01-24

**Authors:** Shu-Fen Niu, Shu-Fen Kuo, Hsiu-Ting Tsai, Ching-Chiu Kao, Victoria Traynor, Kuei-Ru Chou

**Affiliations:** 1 Post-Baccalaureate Program in Nursing, College of Nursing, Taipei Medical University, Taipei, Taiwan; 2 Department of Nursing, Taipei Medical University-Shuang Ho Hospital, Taipei, Taiwan; 3 College of Nursing, Taipei Medical University, Taipei, Taiwan; 4 Department of Nursing, Wan Fang Hospital, Taipei Medical University, Taipei, Taiwan; 5 School of Nursing, Science Medicine and Health, University of Wollongong, Wollongong, NSW, Australia; 6 School of Nursing, College of Nursing, Taipei Medical University, Taipei, Taiwan; 7 Psychiatric Research Center, Taipei Medical University Hospital, Taipei, Taiwan; University of New South Wales, AUSTRALIA

## Abstract

Nurses who experience workplace violence exhibit compromised care quality and decreased work morale, which may increase their turnover rate. This study explored prevalence of workplace violence, the reaction of victims, and workplace strategies adopted to prevent violence among acute psychiatric settings in northern Taiwan. A cross-sectional study was conducted, which consisted of 429 nurses who completed the Chinese version of the Workplace Violence Survey Questionnaire developed by the International Labor Office, International Council of Nurses, World Health Organization, and Public Services International. The rates of physical and psychological violence were 55.7% and 82.1%, respectively. Most perpetrator of the workplace violence were patients. Most victims responded by instructing the perpetrator to stop, followed by narrating the incident to friends, family, and colleagues. Only 4.9%–12% of the victims completed an incident or accident form, and the main reason for not reporting these violent incidents was the belief that reporting such incidents was useless or unimportant. The major strategies adopted by workplaces to prevent violence were security measures, patient protocols, and training. Institutions should train staff to handle violence, provide a therapeutic environment, simplify the reporting process, and encourage reporting of all types of violence.

## Introduction

Nurses who working in psychiatry environments have a 20 times higher rate of physical violence than working in public health unit [1]. Psychiatric nurses experience all types of patient aggression at higher rates than do medical-surgical nurses [2], with an average of 0.55 violent incidents per bed per month in acute psychiatric settings [3]. In mental health facilities, 83% of nurses reported experiencing at least one form of workplace violence in the preceding 12 months, and approximately 33% and 54% of the victims reported resulting moderate psychological distress and severe psychological distress, respectively [4]. Nursing staff experiencing violence in the workplace has become a workplace safety concern to which health institutions worldwide attach considerable importance. Violence is a critical factor that affects the workplace safety of nursing staff. Therefore, recognizing workplace violence is a pressing occupational concern, particularly for psychiatric nurses.

Myriad causative factors, including patients, environment, and employees, contribute to workplace violence. Patients involuntarily admitted to the hospital because of schizophrenia, alcohol use [5], drug misuse, a history of violence, or hostile-dominant interpersonal styles [6] are more prone to violent behavior. Ward environments that increase the risk of violence include an overloaded or a stressful ward atmosphere [7], crowding, locked units, and a lack of therapeutic activities [8]. Nursing staff factors that increase vulnerability to workplace violence include inadequate social skills, low tolerance [9], impaired well-being, complex nursing work [6], communication restrictions [10], and provocation by patients [11].

As first-line guardians in acute psychiatric settings, nursing staff are in close contact with patients and their families, resulting in numerous interactions; thus, they are at risk of encountering violent incidents. Nursing staff who are exposed to violence experience increased stress, decreased work satisfaction [12] and had adverse long-term health consequences [13]; in addition to negative emotions and adverse effects on the body, spirit, and institution, this reduces care quality and work morale and increases nurse turnover [14, 15]. Magnavita [16] conducted a longitudinal study found that workplace violence and work-related distress is bidirectional. Nurses who suffered workplace violence had high strain at work in the following year; high work strains increase interpersonal conflicts, reduce work efficiency and teamwork [17], which may predictor the occurrence of nonphysical violence in the following year.

The violent perpetrators include external sources (patients and their visitors) and internal sources (co- workers) [18], The perpetrators of physical violence were mainly patients or visitors, minority of cases, the attackers were colleagues. Most of the perpetrators of non-physical violence were colleagues or superiors, followed by patients, and their relatives [1, 17]. Horizontal violence among colleagues is a phenomenon in the nursing profession that create a negative work environment, affects teamwork and compromises patient care [19].

The top management of institutions must act to prevent workplace violence [20]. Institutions must provide assistive devices [21], staff training, adequate nursing manpower, and an environment of care improvements as priority strategies for managing workplace violence [22].

Recent studies on workplace violence within psychiatric settings have been conducted in a single health care institution [11, 12, 15, 23, 24], varied definitions of workplace violence, and used questionnaires that did not cover both physical and psychological aspects. Therefore, this study involved multiple centers focused on nursing staff working in locked acute psychiatric settings. Nursing staff in the acute psychiatric settings of 11 health care institutions in northern Taiwan were examined, and workplace violence in terms of both physical and psychological aspects was explored using the Chinese version of the Workplace Violence Survey Questionnaire developed by the International Labor Office, International Council of Nurses, World Health Organization, and Public Services International (ILO/ICN/WHO/PSI). The findings are provided to health care institutions so that strategies for preventing workplace violence and creating safe working environments can be developed.

### Aims

This study explored 1) the prevalence of physical and psychological violence experienced by nursing staff of acute psychiatric settings, 2) the attackers (include external and internal sources) of nursing staff and actions taken against them, 3) the reactions and follow-up assistance status of victims; and 4) strategies that healthcare organizations have implemented to prevent workplace violence.

## Methods

### Study design

A cross-sectional study was performed to determine the prevalence of workplace violence, the reaction of victims, and workplace strategies of acute psychiatric settings in northern Taiwan.

### Participants

The inclusion criteria were being a registered nurse and full-time nursing staff member working in the acute psychiatric ward of a general or psychiatric hospital with at least 6 months of experience. Those not in contact with patients during their work were excluded from the study. There are 1152 acute psychiatric beds in general hospitals and specialty hospitals in northern Taiwan and have a total population of approximately 500 nurses. The sample size was calculated using G-power calculations with a large-sample z-test model [25] with a medium effect size. Statistical significance was set to α = 0.05, and power was set to 0.85, that at least 430 participants would be required, with an estimated attrition rate of 10–15% of the study participants. In total, 480 questionnaires were distributed.

### Data collection

This study was conducted between August 2014 and May 2015. Data were collected using the Chinese version of the Workplace Violence Survey Questionnaire jointly designed and developed by the ILO/ICN/WHO/PSI (2003), which surveys experiences of physical and psychological violence in the workplace over the preceding 12 months. Before the questionnaires were completed, the researcher explained the purpose of the study to the participants and informed them that the survey was confidential and voluntary. After the participants had completed the questionnaire, the questionnaires were placed in sealed envelopes and stored in a box, which was later collected by the researcher. Participants who decided to opt out during the process could do so at any time. Participants were asked to report if they experienced any discomforting memories after completing the questionnaires, and follow-up counseling was arranged for them.

### Validity and reliability

The Chinese version of the Workplace Violence Survey Questionnaire was designed by two nursing scholars proficient in Chinese and English through a double translation approach (translation and back-translation), The original questionnaire was divided into four parts: 1) personal and workplace data, 2) physical workplace violence, 3) psychological workplace violence (including verbal abuse, bullying/mobbing, sexual harassment, threats, and racial harassment), and 4) health sector employer. The Chinese version of the questionnaire is substantially different from the Taiwanese version in terms of culture, since the item “racial harassment” under the Psychological Workplace Violence in the original questionnaire does not conform to the Taiwanese healthcare occupational culture, it was deleted. The sections related to workplace violent experiences consisted of yes–no questions. Personal and workplace variables included age, marital status, educational level, and clinical setting (e.g., shift work and working experience); workplace violence variables included frequency of violence, types of reactions to violence, and consequences for the perpetrators. Four experts from relevant fields in Taiwan were invited to assess the content validity and suitability of the translation, and the content validity index was 0.88. The test–retest reliability was 0.80 [24].

The questionnaire included physical and psychological violence. Physical violence is defined as the use of physical force against another person or group that results in physical, sexual, or psychological harm and includes beating, kicking, slapping, stabbing, shooting, pushing, biting, and pinching. Psychological violence is defined as the intentional use of power, including the threat of physical force, against another person or group that results in harm to physical, mental, spiritual, morale, or social development and includes verbal abuse, bullying/mobbing, harassment, and threats [26, 27].

### Ethical considerations

After the study was approved by the Institutional Review Board of Shin Kong Wu Ho-Su Memorial Hospital (IRB: 20140503R), written informed consent was obtained from each participant. Participants were assured that their personal information would remain confidential and be used only for academic purposes. Additionally, participants were informed that they could withdraw from the study at any time without negative consequences.

### Statistical analysis

IBM SPSS Statistics 23.0 (SPSS, Chicago, IL, USA) was used for data entry and statistical analyses. Descriptive statistics were presented as the percentage, mean, and standard deviation. Inferential statistics included the chi-squared test and *t*-test. Frequency and percentages were used to quantify of various types of violence in demographics, attacker, incident place, the consequences of the attacker, the response of the victim, and the reasons of unreported events. The chi-squared test was used to assess the association between demographic characteristics and the physical or psychological violence, and Student t-test was used to assess the association between age and the physical or psychological violence. Variables significantly associated with either physical or psychological violence were subsequently modeled in multiple logistic regression to evaluate the associations of individual demographic and workplace of violence, odds ratio (OR) and 95% confidence interval (CI) of acute psychiatric nurses experiencing different types of violence. Statistical significance was defined as *P* < 0.05.

## Results

In total, 480 questionnaires were distributed, and 429 valid samples were collected (a response rate of 89.4%).

### Prevalence of physical and psychological violent episodes experienced by nursing staff in acute psychiatric settings

Of the 429 participants, 88.3% (n = 379) were victims of either physical or psychological workplace violence. Of them, 55.7% (n = 239) had experienced physical violence and 82.1% (n = 352) had experienced psychological violence; of violent incidents, 78.8% (n = 338) were categorized as verbal abuse, 55.5% (n = 288) as bullying/mobbing, 32.4% (n = 139) as sexual harassment, and 24% (n = 103) as threats. During physical violence events, 25.1% (n = 60) of the victims were injured, and only 5% (n = 12) requested leave to rest at home after the incidents. Of those who requested leave, 8 victims requested leave of 1–3 days.

### Personal characteristics of the victims of physical and psychological violence

Most of the study participants were women (91.3%). Their average age was 34.23 ± 8.66 years. Most (61.5%) were single or divorced. In terms of education, most (60.4%) were college graduates. Additionally, most (95.1%) were nursing staff members. Furthermore, 33.3% and 66.7% of the participants had ≤5 years and >5 years, respectively, of work experience in the health sector; 75.3% worked rotating shifts.

We compared the distributions of basic personal and workplace information of participants who experienced various types of violence and observed that the marital status, work experience, and rotating shift work variables were statistically significant (Table 1).

**Table 1:** Demographic characteristics of the victims of physical and psychological violence (in *n*, %) (*N* = 429).

Variables	Physical violence			Psychological violence			Total
	Yes (n = 239, 55.7%)	No (n = 190, 44.3%)	*X*^2^	Yes (n = 352, 82.1%)	No (n = 77, 17.9%)	*X*^2^	
**Sex**	1.37				0.02		
Female	215 (90.0)	177(93.2)		322(91.5%)	70(90.9%)		392(91.4%)
Male	24 (10.0)	13(6.8)		30(8.50%)	7(9.1%)		37(8.6%)
**Marital status**		0.05			11.98***		
Single/divorced	146(61.1%)	118(62.1%)		230(65.3%)	34(44.2%)		264(61.5%)
Married	93(38.9%)	72(37.9%)		122(34.7%)	43(55.8%)		165(38.5%)
**Education**		0.40		3.04		
Graduate school or above	29(12.1%)	27(14.2%)		45(12.8%)	11(14.3%)		56(13.0%)
University/College	146(61.1%)	113(59.5%)		219(62.2%)	40(51.9%)		259(60.4%)
Junior college	64(26.8%)	50(26.3%)		88(25.0%)	26(33.8%)		114(26.6%)
**Position**		1.48		3.54		
Manager	9(3.8%)	12(6.3%)		14(4.0%)	7(9.1%)		21(4.9%)
Staff	230(96.2%)	178(93.7%)		338(96.0%)	70(90.9%)		408(95.1%)
**Working experience (years)**			0.35	26.47***			
>15	60(25.1%)	46(24.2%)		72(20.5%)	34(44.2%)		106(24.7%)
>10~≤15	50(20.9%)	37(19.5%)		71(20.2%)	16(20.8%)		87(20.3%)
>5~≤10	52(21.8%)	41(21.6%)		89(25.3%)	4(5.2%)		93(21.7%)
≤5	77(32.2%)	66(34.7%)		120(34.1%)	23(29.9%)		143(33.3%)
**Work in rotating shifts**	4.16*				10.25***		
Yes	189(79.1%)	134(70.5%)		276(78.4%)	47(61.0%)		323(75.3%)
No	50(20.9%)	56(29.5%)		76(21.6%)	30(39.0%)		106(24.7%)
	Mean (SD)	Mean (SD)	t	Mean (SD)	Mean (SD)	t	Mean (SD)
Age	34.18(8.47)	34.28(8.91)	0.12	33.46(7.94)	37.73(10.78)	12.49***	34.23(8.66)

Note

*p<0.05

**p<0.01

***p<0.001

The multiple logistic regression analysis revealed that when the group with >15 years of working experience was used as the reference, nurses with work experience of 5–10 years had an increased risk of psychological violence (OR = 7.48; 95% CI = 2.47–22.68). Those who worked rotating shifts had an increased risk of physical violence (OR = 1.68; 95% CI = 1.05–2.67) and psychological violence (OR = 1.73; 95% CI = 1.03–3.06) (Table 2).

**Table 2:** Factors associated with different types of violence according to a multiple logistic regression analysis, with odds ratios (ORs) and 95% confidences intervals (CIs).

Variables	Physical violence		Psychological violence	
	OR	95% CI	OR	95% CI
**Marital status**				
Single/divorced	1.0		1.0	—
Married	1.05	.66–1.67	0.54	0.29–1.10
**Working experience (years)**				
>15	1.0		1.0	—
>10~≤15	1.19	0.59–2.39	1.90	0.95–3.79
>5~≤10	1.50	0.64–3.49	7.48***	2.47–22.68
≤5	1.841	0.65–5.15	1.42	0.67–2.99
**Work in rotating shifts**				
No	1.0	—	1.0	—
Yes	1.68*	1.05–2.67	1.73*	1.0–3.06
**Age**	0.98	0.94–1.03	0.97	0.92–1.03

Note

*p<0.05

**p<0.01

***p<0.001

### Attackers and consequences for the perpetrator

Most of the perpetrators, of physical or psychological violence, were patients (overall n = 369, 97.4%), followed by patients’ family members. Most violent incidents occurred within health care institutions (overall n = 345, 91.0%). In terms of consequences for perpetrators, most received verbal warnings (overall n = 180, 47.5%), followed by no action (overall n = 95, 25.1%) (Table 3).

**Table 3:** Frequency distributions (in *n*, %) for different types of violent attackers, incident took place, and consequences for the perpetrator.

Variables	Physical violence	Psychological violence (*N* = 352)			
		VA	BM	SH	Threats
	*n* = 239	*n* = 338	*n* = 288	*n* = 139	*n* = 103
**Attacker**					
Patient/client	233 (97.4)	308 (91.1)	123 (42.7)	135 (97.1)	87 (84.5)
Relative of a patient /client	3 (1.3)	126 (37.3)	108 (37.5)	16 (11.5)	24 (23.3)
Staff member	3 (1.3)	28 (8.3)	27 (9.4)	2 (1.4)	7 (6.8)
Management/supervisor		20 (5.9)	15 (5.2)	0 (0.0)	4 (3.9)
Other		12 (3.6)	17 (5.9)	1 (0.7)	9 (8.8)
**Incident took place**					
Inside a health institution or facility	223 (93.3)	331(97.9)	282 (98.0)	136 (97.9)	96 (93.2)
Outside (on way to work/ health visit/ returning home)	16 (6.7)	7 (2.1)	6 (2.0)	3 (2.1)	3 (2.9)
**Consequences for the attacker (multiple answers possible)**					
None	80 (33.5)	92 (27.2)	72 (25.0)	17 (12.2)	19 (18.4)
Verbal warning issued	101 (42.3)	196 (58.0)	161 (55.9)	87 (62.6)	53 (51.5)
Don't know	52 (21.8)	9 (2.7)	8 (2.8)	11 (7.9)	11 (10.7)
Care discontinued	2 (0.8)	10 (3.0)	10 (3.5)	7 (5.0)	6 (5.8)
Reported to police	2 (0.8)	6 (1.8)	8 (2.8)	4 (2.9)	3 (2.9)
Aggressor prosecuted	2 (0.8)	2 (0.6)	0 (0.0)	1 (0.7)	0 (0.0)
Other	0 (0)	23 (6.8)	29 (10.1)	18 (12.9)	15 (14.6)

*Note*: VA, verbal abuse; BM, bullying/mobbing; SH, sexual harassment.

### Responses of victims and support provided

In terms of response to physically and psychological violent incidents, most of the victims instructed the attacker to stop (overall n = 260, 68.6%), followed by narrated the incident to friends and family (overall n = 230, 60.7%) and colleagues (overall n = 221, 58.3%). In terms of response to psychologically violent incidents, “engaging in self-defense” accounted for 57.7% (n = 203) of cases (Table 4). The main reasons for not reporting violent incidents was that reporting the incident was considered useless (overall n = 175, 46.2%) or unimportant (overall n = 157, 41.4%) (Table 4). Counseling, the opportunity to speak about/report it, and other support were provided by employers or supervisors in 87% (n = 208), 87.9% (n = 210), and 75.7% (n = 181) of the physically violent cases and overall 59.4% (n = 209), 71.0% (n = 250), and 46.9% (n = 165) of the psychologically violent cases, respectively.

**Table 4:** Frequency distributions (in %) of responses to the incident and reasons for not reporting the incident (multiple answers possible).

	Physical violence	Psychological violence (*n* = 352)			
Variable		VA	BM	SH	Threats
	*n* = 239	*n* = 338	*n* = 288	*n* = 139	*n* = 103
**Response to the incident**					
Took no action	30 (5.2)	40 (11.8)	43 (14.9)	21 (15.1)	28 (27.2)
Told the person to stop	107 (18.6)	191 (56.5)	164 (56.9)	75 (54.0)	49 (47.6)
Told friends/family	98 (17.0)	178 (52.7)	153 (53.1)	61 (43.9)	48 (46.6)
Reported it to a senior staff	98 (17.0)	104 (30.8)	119 (41.3)	51 (36.7)	31 (30.1)
Told a colleague	68 (11.8)	171 (50.6)	145 (50.3)	68 (48.9)	43 (41.7)
Engaged in self-defense actions	65 (11.3)	149 (44.1)	129 (44.8)	61 (43.9)	40 (38.8)
Completed an incident form	69 (12.0)	32 (9.5)	31 (10.8)	11 (7.9)	5 (4.9)
Tried to pretend it never happened	8 (1.4)	47 (13.9)	36 (12.5)	21 (15.1)	18 (17.5)
Sought help from an association	17 (3.0)	14 (4.1)	17 (5.9)	6 (4.3)	2 (1.9)
Sought counseling	3 (0.5)	16 (4.7)	9 (3.1)	7 (5.0)	5 (4.9)
Transferred to another position	3 (0.5)	5 (1.5)	4 (1.4)	2 (1.4)	0 (0.0)
Pursued prosecution	1 (0.2)	8 (2.4)	6 (2.1)	1 (0.7)	2 (1.9)
Completed a compensation claim	2 (0.3)	3 (0.9)	2 (0.7)	0 (0.0)	0 (0.0)
Sought help from the union	0 (0.0)	2 (0.6)	0 (0.0)	0 (0.0)	0 (0.0)
Other	7 (1.2)	7 (2.1)	3 (1.0)	3 (2.2)	1 (1.0)
**Reasons for not reporting or telling others about the incident**					
	*n* **= 212**	*n* **= 310**	*n* **= 262**	*n* **= 132**	*n* **= 99**
Not important	79 (32.0)	104 (30.8)	86 (29.9)	40 (28.8)	34 (33.0)
Useless	64 (25.9)	131 (38.8)	126 (43.8)	65 (46.8)	45 (43.7)
Afraid of negative consequences	52 (21.1)	49 (14.5)	50 (17.4)	20 (14.4)	14 (13.6)
Felt ashamed	13 (5.3)	13 (3.8)	10 (3.5)	8 (5.8)	7 (6.8)
Felt guilty	8 (3.2)	6 (1.8)	7 (2.4)	1 (0.7)	1 (1.0)
Did not know to whom to report	2 (0.8)	12 (3.6)	14 (4.9)	5 (3.6)	10 (9.7)
Other	29 (11.7)	28 (8.3)	22 (7.6)	10 (7.2)	5 (4.9)

*Notes*: VA, verbal abuse; BM, bullying/mobbing; SH, sexual harassment.

### Workplace violence prevention strategies

In total, 91.4% of participated their institutions had established reporting measures for violence, and 83.2% encouraged victims to report incidents during the execution of nursing service. In terms of strategies adopted by institutions to prevent violence, 79.0% of participants had “security measures” (e.g., guards, alarms, and portable telephones), followed by “patient protocols” (e.g., control and restraint procedures, transportation, medication, activity programming, and access to information; 60.8%) and “training” (e.g., workplace violence–coping strategies, communication skills, conflict resolution, and self-defense; 58.5%) (Table 5).

**Table 5:** Workplaces have already taken measures to prevent violence (multiple choices) (*N* = 429).

Measure	*n* (%)
Security measures (e.g., guards, alarms, and portable telephones)	339 (79.0)
Patient protocols (e.g., control and restraint procedures, transport, medication, activity programming, and access to information)	261 (60.8)
Training for prevent workplace violence.	251 (58.5)
Improvement of surroundings (e.g., lighting, noise, cleanliness, privacy)	224 (52.2)
Patient screening (to record and be aware of previous aggressive behavior)	207 (48.3)
Restricted exchange of money at the workplace (e.g., patient fees)	124 (28.9)
Special equipment or clothing (e.g., uniforms or the absence of uniforms)	105 (24.5)
Restricted public access.	103 (24.0)
Reduced periods of working alone.	97 (22.6)
Investment in human resource development.	72 (16.8)
Check-in procedures for staff (especially for home care).	39 (9.1)
Increased staff numbers.	37 (8.6)
Changed shifts or rotations (i.e., working times).	27 (6.3)
None of the above.	9 (2.1)

## Discussion

This study determined that the 12-month prevalence of violence experiences was 88.3%, which is similar to that determined by a study on mental health service employees in Australia (83% of whom were exposed to workplace violence) by Tonso et al. [4]. In the present study, 55.7% of the nurses had experienced physical violence, and 82.1% had experienced psychological violence. These rates are similar to those determined in an integrative study of 38 countries by Spector, Zhou, & Che [28] in which the rates of physical violence and psychological violence in psychiatric settings averaged 55% and 72.8%, respectively.

Those who worked rotating shifts were more likely to experience violence than were those who did not; this finding is consistent with that of Jiao et al. [29]. Nurses working rotating shifts were more likely to experience physical and psychological violence than were those working fixed day shifts. Occupational Safety and Health Administration (OSHA) [30] stated that working alone in a facility is a risk factor for violence. Night shifts are usually less nursing staffing, and Cheng and Cheng [31] indicating that night shifts and rotating shift workers have lower job control, reduce workplace justice, and higher job insecurity. Working shifts was considered high risk for exposure to violence. It is recommended to implement an appropriate security and surveillance facilities such as metal detector and emergency alarming system in the workplace to reduce possible violence. Furthermore, employers must conduct risk assessment tests to measure the risk of working at night shift and have a sufficient number of nursing manpower available to enable nurses to take breaks and eat meals.

In this study, nurses with work experience of 5–10 years were at increased risk of psychological violence. This finding was consistent with the research of Chen, Lv [32], in their study, N2 –N4 nurse were risk factors related to non-physical violence. Part of the reason were the allocation of work content, where intensive and technical work were assigned among senior nurses. In Taiwan, the nursing staff were categorized into five levels of the clinical ladder (N, N1, N2, N3, N4). At N, N 1 performs patient/case basic care, and N2-N4 requires to perform holistic care for critically ill patients/cases. Nurses with 5–10 years of working experience (mostly N2-N4), are likely to be assigned patients with higher disease severity who are difficult to handle, thereby exposing them to more stressful situations, which might result in them having greater exposure to psychological violence than more junior nurses.

Most perpetrators in the present study were patients, which was consistent with the findings of numerous studies [23, 33, 34]. Nursing staff directly provide patient care; thus, they interact more frequently with patients and their families. Violence can also be caused by patients having insufficient information or misunderstanding the information related to their overall disease, inspection, and treatment results. This study found that 14.2%–14.6% of verbal abuse and bullying/mobbing originated from colleagues and management. Horizontal violence is notable because verbal abuse and bullying from superiors and coworkers reduces enthusiasm and thereby nurses’ job satisfaction and morale, as well as health [18]. OSHA recommends that management and employees participate in the creation and operation of workplace violence prevention programs to effectively prevent violence [30], establish a positive organizational psychosocial safety climate [35] and regular survey of workplace violence [18] to reduce the incidence of internal violence.

Psychological violence was more prevalent than physical violence, with verbal abuse occurring in 78.8% of cases, with 91.1% of the perpetrators being patients. In this study, all locked acute psychiatric setting limited patients from freely entering and leaving the ward. Because of restrictions and disease characteristics, patients were often unsatisfied, which generally resulted in a greater frequency of verbally violent. It is recommended that the principles of trauma-informed care model used to provide consistent, honest and compassionate relationship, to provide choice and control for patients [36] and mitigating the atmosphere of violence.

Concerning nursing staff’s responses to violent incidents, this study determined that instructing the perpetrator to stop was the commonest response, followed by telling friends and family, and then colleagues. The participants needed to release their emotions after exposure to violence [37]; thus, they discussed the incidents and shared their feelings with family members, peers, and friends. We ascertained that 91.4% of all institutions in this study had established reporting measures for violence, and 83.2% encouraged victims to report violent incidents. However, we also ascertained that of those who had experienced violence, only 12% reported physically violent incidents, and only 4.9%–10.8% reported psychological violence. The number of reported psychologically violent incidents was considerably lower than the actual number of occurrences. This finding, similar to that of Duncan et al. [38], indicated that 70% of nurses experienced violent incidents but did not report them. In this study, the main reason for not reporting incidents was considering reporting them useless and unimportant. The psychological and emotional damage caused by psychological violence cannot be detected from one’s appearance and is difficult to perceive visually. Furthermore, the medical culture emphasizes service quality and customer orientation, believing in the concept of “the customer comes first.” Additionally, when patients have a cognitive impairment, such as dementia or brain damage and a reduced ability to control themselves and to understand the consequences of their actions, nurses often do not consider what contributed to any deliberate intentional or planned behavior [39], and most nurses do not want to hold the perpetrator liable. Coupled with their busy workloads, the cumbersome reporting process and the belief that reporting incidents will not change the current situation contributed to the low reporting rate. Moreover, the willingness of managers to defend the nurses will affect the frequency of workplace violence reports [39]. It is advisable to monitor and educate managers response better to victims and establishing a streamlined incident reporting process to increase reporting rate and uniform violence reporting system to encourage reporting. In incidents of psychological violence, this study established that 38.7%–44.8% of the victims reported engaging in self-defense; however, only half of those who experienced psychological violence received counseling. Workplace violence can affect work enthusiasm and performance, reduce job satisfaction, and affect the retention of nursing staff [40]. Therefore, should prioritize the psychological healing and provide trauma crisis counseling to help the victims of psychological violence.

In terms of the strategies adopted by institutions to prevent violent incidents, only 58.5% of the respondents had received training in violence management. Education and training are key elements of workplace violence protection [30, 41], the employers need to provide continuing education and training for all employees to increase their awareness of potential violence risks. The implementation of multi-level actions can effectively prevent workplace violence and reduce the frequency of attacks [41]. Therefore, employees should be trained to regulate their emotions, actively advocating nurses’ handling and communication skills, establishing contingency measures and guidelines, and conducting analyses, reviews, advocacy, sharing, and learning about violent incidents should be encouraged to provide safe and reliable working environments.

### Strengths and limitations

This study examined nursing staff at the acute psychiatric settings of 11 health care institutions in northern Taiwan; thus, the results have greater generalizability than would those derived from a single institution. The questionnaires used included both physical and psychological aspects and had clear definitions of workplace violence.

This study did not assess individual nurse–patient ratios or environmental factors at each health institutions but selected for the acute stage of patients that they accepted and treated. Future studies should include the workload of nursing staff because it affects workplace violence. This study examined acute psychiatric settings in northern Taiwan; thus, its findings might be inapplicable to situations in rural areas or long stay psychiatric settings. This study retrospectively surveyed the prevalence of violence that occurred within the preceding 12 months; therefore, recall bias is possible.

## Conclusions

During the previous 12 months, over half of acute psychiatric nurses had experienced physical violence and four-fifths had experienced psychological violence. The majority of attackers were patients, followed by family members of patients. The main method of handling attackers was verbal warnings. For actions taken or responses of the victims, the majority “told the person to stop”, followed by “told friends/family” and “told a colleague”. Only 12% reported physically violent incidents, and only 4.9%–10.8% reported psychological violence, the primary reasons for not reporting or telling about violent incidents were “useless”, “not important”. Most institutions encourage victims to report violent incidents, but the number of reported violent incidents was considerably lower than the actual number of occurrences, institutions should be no punishing or labeling staff who report incidents, establishing a streamlined incident reporting process to increase reporting rate. The occurrence of workplace violence can affect the physical and psychological health of victims. We recommend that institutions provide multilevel actions to reduce workplace violence and create safe working environments.
